# Successful treatment of hidradenitis suppurativa and Crohn's disease with combined guselkumab and apremilast

**DOI:** 10.1111/dth.15743

**Published:** 2022-08-12

**Authors:** Manuel Agud‐Dios, Jorge Arroyo‐Andrés, Carmen Rubio‐Muñiz, Concepción Postigo‐Lorente

**Affiliations:** ^1^ Department of Dermatology Hospital Universitario 12 de Octubre Madrid Spain

Dear Editor,

Between 12 and 18% of patients with Crohn's disease (CD) have hidradenitis suppurativa (HS) and clinical and radiological differentiation between both entities when affecting the perianal area can be extremely challenging. The presence of HS is associated with an earlier onset, more aggressive CD, treatment failure, more surgical requirements and lack of response to anti TNFalfa antibodies.^1^, ^2^

A 38‐year‐old man was diagnosed in 2006 of extensive ileocolonic and perianal CD and in 2008 of HS Hurley stage III affecting inguinoscrotal (Figure 1, left), perianal and facial area. During the following years, both diseases had very aggressive course, requiring 25 surgical interventions (including multiple drainages of abscesses, scrotectomy, and colostomy), multiple cycles of antibiotics, corticosteroids, and azathioprine. Biological treatment included infliximab, adalimumab (both without response) and ustekinumab (at doses prescribed for CD, improving only during induction phase and without control of perianal disease). Tissue expression of interleukins revealed high levels of IL‐1b but neither with anakinra nor canakinumab a substantial clinical benefit was obtained.

**FIGURE 1:**
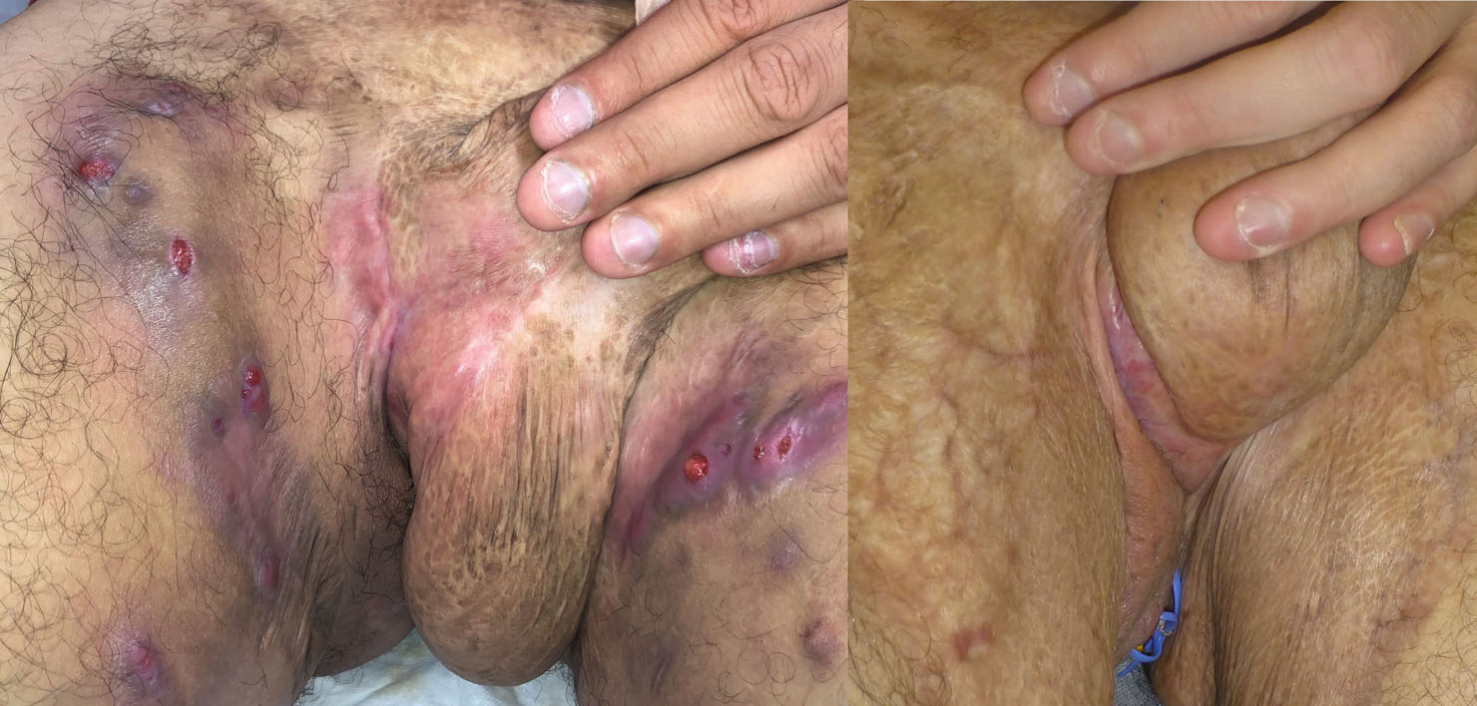
Clinical aspect of inguinal area at baseline before guselkumab was started, showing numerous abscesses and draining fistulae associated with hypertrophic scarring. The second image was taken after 6 months of combined guselkumab and apremilast treatment, showing complete resolution of inflammatory burden

From 2019 to date the patient is under treatment with apremilast 30 mg bid and dapsone with improvement of facial lesions but without significant perianal or inguinal response. In 2020, subcutaneous guselkumab (100 mg monthly) was added and after 3 months, IHS4, DLQI, and visual analogue scale (VAS) for pain have changed, respectively, from 13, 10, and 7 before treatment, to 1, 0, and 0. After 2 years of treatment no inflammatory lesions have reappeared (Figure 1, right), inflammatory markers in blood have normalized and current MRI studies only reveal fibrosis. Dapsone has been tapered to 50 mg per day without relapse. CD has also remained controlled without relapses or significant activity in MRI studies and colonoscopy.

Treatment in CD and HS is evolving rapidly and there are increasing therapeutical options, however, severe forms of both diseases are still often treatment‐refractory with dramatic impact in quality of life.^3^

Excellent response of psoriasis with guselkumab has led to its increasing use for the treatment of HS but to date only few case series and reports have been published. A systematic review in 2020 reported 16 patients with severe HS treated with guselkumab and found that up to 40% of patients with prior failure to other biologics improved after guselkumab was started and that clinical improvement was noted after 12 weeks of treatment.^4^ Previous treatment with ustekinumab and anti‐IL17 antibodies was associated with poorer response.

In CD, selective IL23‐inhibition with intravenous guselkumab (200–1200 mg monthly) has been recently evaluated in a randomized trial against placebo and achieved better clinical and endoscopic outcomes at week 12.^5^ However, patients treated previously with ustekinumab were excluded.

To date, only three other case reports have been published in which guselkumab is used for coexisting CD and HS, all with positive outcomes.^6^, ^7^, ^8^ Additional benefit can be expected in patients who also have psoriasis.

Apremilast has also been used recently for treatment‐refractory HS. In a clinical trial against placebo (*n* = 20), patients treated with apremilast achieved HS clinical response with statistical significance and fewer abscesses, nodules, itch or pain during follow‐up.^9^

Our patient did not respond with apremilast and dapsone in the perianal area and groin and it was after guselkumab was added that an excellent overall control of both HS and CD could be achieved. No other reports have been published of combined treatment with guselkumab and apremilast in this setting. Although more robust evidence is lacking and preliminary trials of guselkumab in hidradenitis show only moderate benefit, our case had excellent response with this combination and could be of substantial usefulness in patients with concomitant HS and CD.

## AUTHOR CONTRIBUTIONS

*Full access to all of the data in the study and take responsibility for the integrity of the data and the accuracy of the data analysis*: Manuel Agud de Dios. *Study concept and design*: Manuel Agud de Dios. *Acquisition, analysis, and interpretation of data*: Manuel Agud de Dios, Jorge Arroyo‐Andrés, Carmen Rubio‐Muñiz, Concepción Postigo‐Lorente. *Drafting of the manuscript*: Manuel Agud de Dios, Concepción Postigo‐Lorente. *Critical revision of the manuscript for important intellectual content*: Concepción Postigo‐Lorente. *Statistical analysis*: None. *Obtained funding*: None. *Administrative, technical, or material support*: Jorge Arroyo‐Andrés, Carmen Rubio‐Muñiz. *Study supervision*: Concepción Postigo‐Lorente.

## CONFLICT OF INTEREST

None, by any of the authors.

## CONSENT STATEMENT

Written consent for publication by the patient was obtained.

## Data Availability

The data that support the findings of this study are available from the corresponding author upon reasonable request.
